# Tumor-Associated Macrophages: Therapeutic Targets for Skin Cancer

**DOI:** 10.3389/fonc.2018.00003

**Published:** 2018-01-23

**Authors:** Taku Fujimura, Yumi Kambayashi, Yasuhiro Fujisawa, Takanori Hidaka, Setsuya Aiba

**Affiliations:** ^1^Department of Dermatology, Tohoku University Graduate School of Medicine, Sendai, Japan; ^2^Department of Dermatology, University of Tsukuba, Tsukuba, Japan

**Keywords:** tumor-associated macrophages, immunosuppression, M2 polarization, chemokines, angiogenetic factors, regulatory T cells

## Abstract

Tumor-associated macrophages (TAMs) and regulatory T cells (Tregs) are significant components of the microenvironment of solid tumors in the majority of cancers. TAMs sequentially develop from monocytes into functional macrophages. In each differentiation stage, TAMs obtain various immunosuppressive functions to maintain the tumor microenvironment (e.g., expression of immune checkpoint molecules, production of Treg-related chemokines and cytokines, production of arginase I). Although the main population of TAMs is immunosuppressive M2 macrophages, TAMs can be modulated into M1-type macrophages in each differential stage, leading to the suppression of tumor growth. Because the administration of certain drugs or stromal factors can stimulate TAMs to produce specific chemokines, leading to the recruitment of various tumor-infiltrating lymphocytes, TAMs can serve as targets for cancer immunotherapy. In this review, we discuss the differentiation, activation, and immunosuppressive function of TAMs, as well as their benefits in cancer immunotherapy.

## Introduction

Tumor-associated macrophages (TAMs) and regulatory T cells (Tregs) are significant components of the tumor microenvironment (1, 2). TAMs express immune checkpoint modulators [e.g., B7 family, B7-homolog family including programmed death ligand 1 (PD-L1)] (3) that directly suppress activated T cells. In addition, TAMs produce various chemokines that attract other immunosuppressive cells such as Tregs, myeloid-derived suppressor cells (MDSCs), and type 2 helper (Th2) T cells, which maintain the immunosuppressive factors of the tumor microenvironment (1, 2, 4). Moreover, TAMs also produce matrix metalloproteinases (MMPs), which play critical roles in tissue remodeling associated with various physiological processes such as morphogenesis, angiogenesis, tissue repair, local invasion, and metastasis (1, 5, 6). TAMs have been detected in various skin cancers such as melanoma, squamous cell carcinoma (SCC), extramammary Paget’s disease (EMPD), Merkel cell carcinoma, basal cell carcinoma, and mycosis fungoides (MFs) (1, 2, 7–15) (Table 1). Because the stromal factor on each cancer stem cell is an important factor for TAM stimulation, leading to the induction of specific TAM phenotypes, investigating the immunomodulatory stromal cells in the tumor microenvironment is important for establishing the appropriate immunotherapy for each type of cancer (1, 8, 9, 16, 17). In addition, it may be possible to repolarize TAMs into anti-tumor macrophages, such as M1-phenotype macrophages, to suppress tumor progression by modifying the profiles of tumor-infiltrating lymphocytes (TILs) (7, 18, 19). Thus, TAMs could be a target for immunotherapy in skin cancers (1, 2). In this review, we discuss the differentiation, activation, and immunosuppressive function of TAMs, as well as their benefit in cancer immunotherapy.

**Table 1:** Tumor-associated macrophages in skin cancer: mouse and human models.

Cancer species	Mouse (reference)	Human (reference)	Depletion	Reprogrammed	Biomarkers
Malignant melanoma	(3, 7, 13, 19, 20, 22, 39, 51, 62, 63, 64, 65)	(7, 35, 59, 60)	(13, 65)	(5, 19, 20, 22, 35, 39)	(3, 59, 60, 61)
Cutaneous squamous cell carcinoma	(23, 24, 32)	(11, 12, 34)	(23)	(24, 32)	(11, 12)
Merkel cell carcinoma	–	(14, 36)			(14, 36)
Extramammary Paget’s disease	–	(8, 17)		(17)	(8)
Basal cell carcinoma	(26)	(15)	(26)		(15)
Dermatofibrosarcoma protuberans	–	(5)			(5)
Cutaneous T cell lymphoma	(25)	(9, 18, 28, 29, 30, 31, 57)	(25)	(18, 57)	(9, 28, 29, 30)

## Differentiation and Activation of TAMs in Tumors

**Figure 1:**
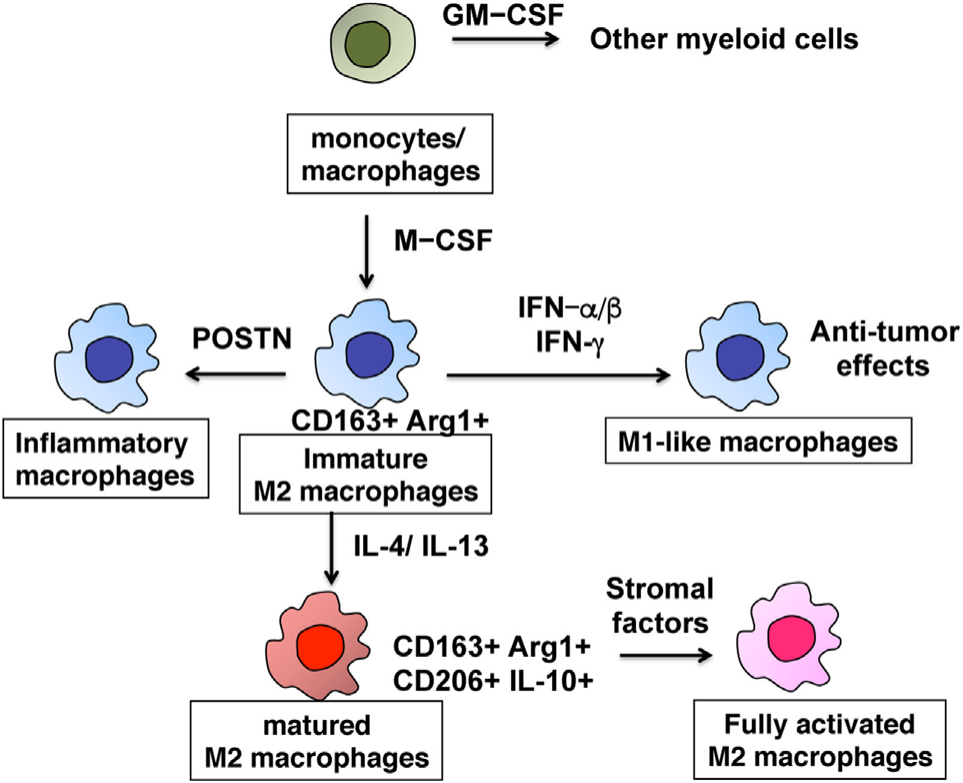
Differentiation of M2-polarized tumor-associated macrophages. The multiple steps of the development of monocytes into fully functional macrophages.

Tumor-associated macrophages are characterized by their heterogeneity and plasticity, as they can be functionally reprogrammed to polarized phenotypes by exposure to cancer-related factors, stromal factors, infections, or even drug interventions (1, 2, 7, 9, 11, 17, 19). Because TAMs sequentially differentiate from monocytes into functional macrophages through multiple steps, they have heterogeneity and plasticity in cancer (Figure 1). Monocytes recruited from the circulation differentiate into tissue macrophages by macrophage colony-stimulating factor (M-CSF), and are primed with several cytokines such as interferon gamma (IFN-γ), interleukin 4 (IL-4), and IL-13 (2). Thereafter, macrophages change their functional phenotype in response to environmental factors or even tumor-derived protein stimulation (2, 8, 17). In skin cancer, for example, targeting the M-CSF receptor with anti-CSF short interfering RNA (siCD115) in TAMs led to modulation of the TIL profile, resulting in growth suppression of B16 melanoma *in vivo* (20). In the second phase of priming, type I IFN (IFN-α, IFN-β) and type II IFN (IFN-γ) modulate the production of chemokines from TAMs, suggesting that these cytokines repolarize TAMs in several skin cancers (7, 18). Cancer stromal factors such as soluble receptor activator of nuclear factor kappa-B ligand (RANKL) derived from cancer cells could be a third mode of stimulation that activates mature M2 macrophages to produce a series of chemokines that recruit immunosuppressive cells such as Tregs and Th2, leading to maintenance of the tumor microenvironment (8, 10, 17). These reports suggest that each of these three differentiation steps could serve as a target for immunotherapies.

## Roles of TAMs in Maintaining the Immunosuppressive Microenvironment

### Chemokines from TAMs Determine the Immunological Microenvironment in Tumors

Chemokines play crucial roles in determining the profiles of TILs in the tumor microenvironment, and the profiles of chemokines from TAMs are determined by stromal factors of each skin cancer (1). For example, immune cells in the tumor microenvironment determine the aggressiveness of melanoma (21). In metastatic melanoma, periostin (POSTN) is expressed in the region surrounding melanoma cell nests in metastatic melanoma lesions that develop at the wound site (16). In addition, TAMs are prominent in the tumor stroma in melanoma (7, 19, 22), and POSTN stimulates CD163^+^ macrophages to produce several specific cytokines including Treg-related chemokines [chemokine ligand 17 (CCL17), CCL22] (9). Because CCL17 and CCL22 from TAMs attracts Tregs to the tumor site in melanoma (7, 21, 22), repolarization of TAMs by immunomodulatory reagents such as IFN-β and imiquimod are useful for suppressing tumor growth in melanoma (7, 22). The downregulation of CCL22 production was also observed in B16F10 melanoma mouse treated with classical cytotoxic anti-melanoma drugs such as dacarbazine, nimustine hydrochloride, and vincristine, all of which have been used in the adjuvant setting for advanced melanoma for the last 30 years (19). Other reports have suggested that a series of chemokines (CCL17, CXCL10, CCL4, and IL-8) in cerebrospinal fluid may be useful for predicting brain metastasis in melanoma patients (21). Together, these reports suggest the significance of chemokines from TAMs that can be induced by POSTN in the tumor stroma to induce melanoma-specific TILs in patients with melanoma.

Tumor-associated macrophages in non-melanoma skin cancer also secrete an array of chemokines in lesional skin to regulate the tumor microenvironment (1). In EMPD, for example, soluble RANKL released by Paget cells increases the production of CCL5, CCL17, and CXCL10 from RANK^+^ M2 polarized TAMs (8, 10, 17), suggesting that Paget cells can determine the immunological microenvironment by the stimulation of TAMs. The results of this study led to the hypothesis that denosumab, a full human monoclonal antibody for RANKL, has therapeutic effects in invasive EMPD. In cutaneous squamous cell carcinoma (cSCC), according to its heterogeneity of differentiation of cancer cells, TAMs in cSCC heterogeneously polarized from M1 to M2 (11). Indeed, Petterson et al. (11) reported that CD163^+^ TAMs not only express CCL18 (11), an M2 chemokine involved in remodeling of the tumor microenvironment but are also colocalized with phosphorylated signal transducer and activator of transcription 1 (11), suggesting the heterogeneous activation states of TAMs. Although the exact stimulator of cSCC is unknown, the depletion of TAMs such as antibody-mediated depletion (e.g., anti-CSF1R Ab) or bisphosphonate could be a useful therapy for unresectable cSCC (23–26).

Not only solid tumors but also hematopoietic malignancies in the skin contain CD163^+^ TAMs (25, 27–29), which produce chemokines that direct to specific anatomic sites to form metastases (25). Indeed recently, Wu et al. (9) used a human xenograft CTCL cell model to demonstrate that chemokines from TAMs play crucial roles in tumor formation in MF lesions. In another report, it was shown that the cancer stroma of MF containing POSTN and IL-4 might stimulate TAMs to produce chemokines that correlate with tumor formation in MF (25), and that chemokines from TAMs can be modified by immunomodulatory agents such as IFN-α and IFN-γ, leading to their therapeutic effects (18). Furthermore, CCL18 produced by TAMs in MF at the invasive margin of the tumor promote the recruitment of CTCL cells, leading to cancer progression (30). These reports suggest the significance of chemokines from TAMs for the development of CTCL.

### Direct Suppressive Function of TAMs

Immunomodulatory costimulatory molecules, such as B7 homologs, play representative roles in the direct cell-mediated suppressive mechanism of TAMs. Recently, several reports have suggested that the expression of PD-L1 (also known as B7H1) in TAMs is necessary for antigen-specific tolerance induction (1, 3, 31) in tumor-bearing hosts. For example, the expression of PD-L1 on TAMs is augmented by autocrine IL-10 from M2-polarized TAMs stimulated by specific antigens (31). Another report showed that the decrease of IL-10 in MDSCs led to the downregulation of PD-L1 expression in MDSC in a mouse melanoma model (3). Linde et al. (32) reported that IL-10-polarized TAMs into M2 phenotypes in the presence of IL-4 and vascular endothelial growth factor A (VEGF-A) in cSCC. These reports suggest that IL-10 upregulates PD-L1 expression on TAMs, inducing immunosuppression in the tumor microenvironment in the skin. Arginase 1 is one of the key factors for the suppressive function of TAMs. Its expression is widely detected in immature and functional M2 macrophages (1, 8, 17), leading to suppression of T cell activity by l-arginine catabolism (33). Indeed, CD163^+^ TAMs expresses arginase 1 in several skin cancers such as EMPD and SCC (8, 34). More recently, Pico de Coaña et al. (35) reported the additional immunomodulatory effects of ipilimumab on granulocytic MDSCs, which are circulating macrophages in tumor-bearing hosts, suggesting the crosstalk between Tregs and granulocytic MDSCs through the CTLA4/B7 homolog pathway and the significance of the direct suppressive function of TAMs (35).

### Angiogenetic Factors from TAMs

Tumor-associated macrophages produce angiogenetic factors such as VEGF, platelet-derived growth factor, and transforming growth factor β, or by expressing MMPs to induce neovascularization (10, 28, 32, 36–38). Linde et al. (32) reported that VEGF-A augments the recruitment of TAMs at a tumor site by promoting neovascularization in a mouse skin tumor model (32). In a human skin cancer model, Werchau et al. (36) reported that VEGF-C expressed by TAMs contributes to lymphangiogenesis and the progression of Merkel cell carcinoma (36). In angiosarcoma, TAMs express MMP9, which might be a target for amino bisphosphonate (37). Another report suggested that inhibition of the VEGF/VEGF receptor pathway inhibits M2 polarization in TAMs, leading to reduced vascular density and tumor growth in MCA205 mouse sarcoma (38). In addition, more recently, Yamada et al. (39) reported that the expression of MGF-E8 on mesenchymal stromal cells plays crucial roles in inducing M2 macrophage polarization, leading to suppression of tumor growth by the reduction of VEGF expression in TAMs in B16F10 melanoma. These reports indicate the significance of VEGF produced by M2 macrophages in tumor progression, and show that both VEGF and MMPs are key markers for M2 macrophages in skin cancers (11, 40, 41). For example, in a melanoma model, osteopontin signaling promoted macrophage recruitment by the secretion of prostaglandin E2 and MMP-9 from TAMs, leading to angiogenesis and tumor progression (41). These reports suggest that MMPs play crucial roles in tumor progression. MMPs can also be produced by TAMs upon stimulation of stromal proteins in skin cancer (9, 10). For example, the stimulation of POSTN augments the production of MMP1 and MMP12 from monocyte-derived immature M2 macrophages (9). Because POSTN is abundant in the tumor stroma of MF and dermatofibrosarcoma protuberans (DFSP) (5, 9), and because substantial numbers of CD163^+^ TAMs have been detected in the POSTN-rich area in the lesional skin of skin tumors (5, 9), the production of MMP1 and MMP12 is prominent in the lesional skin of MF and DFSP. Notably, as reported by Livtinov et al. (42), among the MMPs, only MMP12 is a risk factor for CTCL progression, as determined by transcriptional profiling (42). RANKL is expressed in skin cancers of apocrine origin such as EMPD and apocrine carcinoma (8, 37), and is released in its soluble form. Because monocyte-derived M2 macrophages produce MMP1 and MMP25 by RANKL stimulation, TAMs in skin cancer of apocrine origin produce MMP1 and MMP25 at the tumor site (37). These reports suggest that TAMs stimulated by tumor stromal factors play roles in the carcinogenesis of these skin cancers, and might be targets for molecular-targeted therapy in the future.

## Clinical Benefits of TAMs

### The Effects of Anticancer Drug for TAMs

Because TAMs comprise the immunosuppressive microenvironment at the tumor site, they may be optimal therapeutic targets in cancer (1, 2, 4, 43–46). For example, Rogers et al. (44) reported the immunomodulatory effects of bisphosphonate on TAMs in patients with breast and prostate cancers upon the repolarization of TAMs into tumoricidal macrophages (44). More recently, several reports have also focused on the immunomodulatory effects of chemotherapeutic reagents on TAMs (19, 47, 48). For example, a non-cytotoxic dose of paclitaxel decreased MDSCs and even blocked the immunosuppressive potential of MDSCs in a mouse melanoma model (47). More recently, Fujimura et al. (19) reported the immunomodulatory effects of cytotoxic anti-melanoma drugs, dacarbazine, nimustine hydrochloride, and vincristine, on TAMs both *in vitro* and *in vivo* by inhibition of STAT3 signals (19). The authors concluded that their immunomodulatory effects could explain their antitumor effects in postoperative melanoma patients. Peplomycin administered through a superficial temporal artery using an intravascular indwelling catheter, which can cause dose-independent interstitial pneumonia (49), decreased the number of TAMs and Tregs in cSCC on the lips, leading to an increase in the number of immunoreactive cells at the tumor sites (50), and possible autoimmune-like interstitial pneumonia (49, 50). More recently, not only cytotoxic chemotherapeutic drugs but also low molecular weight compounds were reported to co-localize with TAMs at tumor sites. Indeed, Hu-Lieskovan et al. (13) reported that single-agent dabrafenib increased TAMs and Tregs in melanoma, which decreased with the addition of trametinib, leading to the synergistic effects of immune checkpoints inhibitors with dabrafenib and trametinib combination therapy. In another report, the anti-macrophage receptor with collagenous structure was reported to polarize TAMs into proinflammatory phenotypes to induce anti-melanoma immune response in B16 melanomas (51). In addition, Gordon et al. (52) reported that inhibition of PD-1/PD-L1 *in vivo* increased macrophage phagocytosis, reduced tumor growth, and prolonged the survival of macrophages. In another report, increasing expression levels of PD-L1 in TAMs, 2 months after the administration of anti-PD-1 Abs in patients with advanced melanoma, was correlated with the response to immunotherapy (53), suggesting that PD-L1 expression in TAMs could be a biomarker that predicts the effectiveness of anti-PD-1 Ab therapy. Because the anti-PD-1 Abs nivolumab and pembrolizumab are widely used to treat advanced cancer, including melanoma (53), one target of anti-PD-1 Abs in patients with advanced melanoma could be an immunomodulatory effect on TAM, which, in turn, might be correlated with both their effectiveness and the development of adverse events. TAMs produce not only chemokines that directly recruit immunosuppressive cells to the tumor microenvironment but also produce cytokines that stimulate other stromal cells such as fibroblasts to produce chemokines (54, 55). Indeed, Young et al. (54) reported that IL-1β from TAMs stimulate fibroblasts to produce CXCR2 ligand, which plays crucial roles in recruiting granulocytic MDSCs to tumor sites (55, 56). The authors concluded that CXCR2 agonists in combination with anti-CD115 Abs could suppress B16F10 melanoma *in vivo* by inhibiting the recruitment of granulocytic MDSCs and depletion of immature TAMs (56). Interestingly, the antihuman CD115 Ab, emactuzumab, decreased the number of CD163^+^ CD206^+^ M2 macrophages in patients with melanoma by depleting immature TAMs before the IL-4 stimulation phase (57). Together, these reports suggest that anti-CXCR2 agonists in combination with emactuzumab might induce the antimelanoma immune response by reducing the number of M2 polarized TAMs. These reports suggest the significance of assessing the effects of chemotherapeutic drugs on TAMs (13, 19, 47, 49, 50).

### TAMs as a Biomarker for Disease Activity and Adverse Events

As described above, because TAMs produce tumor-specific chemokines by the stimulation of stromal factors, chemokines might serve as biomarkers that reflect disease activity. For example, TAMs produced CCL18 in the lesional skin of CTCL (26), which reflect disease severity and prognosis (58). Immunomodulatory reagents such as IFNs and imiquimod reduce CCL22 from TAMs, leading to the therapeutic effects of them in mouse B16F10 melanoma models (7, 22). CCL5, which induces Th2 cells from naive T cells (59), reflects the cancer stage and disease progression in gastric cancers (60). Another TAM-associated factor, sCD163, could be a useful biomarker for cancer treatment, as it is an activation marker for CD163^+^ tissue macrophages that is present in the serum as a result of proteolytic shedding (61). Serum sCD163 levels increase in autoimmune diseases such as atherosclerosis, rheumatoid arthritis, moyamoya disease, pemphigus vulgaris, and bullous pemphigoid (62–64), and reflect disease activity (61). Therefore, as we previously reported, sCD163 is a possible marker for predicting immune-related adverse events caused by immune checkpoints inhibitors (64, 65). These reports suggested that the production derived from TAMs could be a biomarker for cancer treatment in the future.

## Concluding Remarks

Although several studies have suggested that high numbers of TAMs in tumor-bearing individuals are associated with a poor prognosis, making them useful as prognostic markers in cancer, further studies are needed to quantify their impact in different cancers.

## Author Contributions

FT designed the study. FT, KY, and HT wrote the article. FT, FY, and AS supervised the study.

## Conflict of Interest Statement

The authors declare that the research was conducted in the absence of any commercial or financial relationships that could be construed as a potential conflict of interest.
